# Operational Challenges in Large Clinical Trials: Examples and Lessons Learned from the Gambia Pneumococcal Vaccine Trial

**DOI:** 10.1371/journal.pctr.0010016

**Published:** 2006-07-14

**Authors:** Felicity T Cutts, Godwin Enwere, Syed M. A Zaman, Fred G Yallop

The requirements for Good Clinical Practice (GCP) in clinical trials are well documented [1], and ethical issues are hotly debated [2]. Operational aspects of trials, however, have received far less attention [3], perhaps due to restrictions on journal space for detailing methods. In low resource settings, however, large trials often face many logistical and organizational obstacles, and thus the practical difficulties in running a trial to GCP standards should not be ignored. Here, we describe the main operational challenges to a randomized, double-blind, placebo-controlled trial of the safety and efficacy of pneumococcal conjugate vaccine among over 17,000 infants in the Gambia. The trial began in August 2000, and after the magnitude of the challenges were recognized, a new senior principal investigator (FTC) and project manager (FGY) were recruited, taking up post in June 2001. We summarize here the major lessons learnt in trial implementation in a resource-poor setting.

## Overview of Trial Preparation and Study Methods

The trial was conducted in Upper and Central River Divisions of the Gambia, covering an area of about 5,000 km^2^, bisected by the river Gambia. Government mother-child health (MCH) services are provided at 15 fixed facilities, the two largest being Bansang hospital in the Central River Division and Basse health centre in the Upper River Division, and about 100 additional outreach sites. The Medical Research Council (MRC) main station is in Fajara, 380 km from Basse. The journey between them took 5 hours in 2000 when the trial began, and, due to deteriorating road conditions, 9–10 hours by 2004 when it ended.

Preparatory studies took over 12 years, and included phase I [4] and phase II vaccine trials [5], as well as baseline studies of rates of disease and mortality [6,7], and selection of the best design for the phase III trial [8,9]. Toward the end of this period, the study area was mapped and a household numbering system was devised. Communities and families were informed about the forthcoming trial, through drama performances in large villages, radio spots, distribution of flyers, and community meetings. Unfortunately, we did not evaluate which method was most effective for enhancing community support and understanding of the trial.

The trial was conducted as a partnership between MRC and the Gambia Government (GG), children being recruited and vaccinated at GG MCH clinics and referred through these clinics for investigations. If MCH clinics or outreach visits were cancelled, this would jeopardise both public health services and the research. The trial funders and MRC therefore invested greatly in infrastructure (capital equipment and buildings, laboratories, radiology facilities, cold chain, and transport) for both Basse field station and GG health services.

The study methods, which followed detailed standard operating procedures (SOPs), have already been described in detail [10]. The primary endpoint for the trial was initially all-cause mortality but was later changed to radiologically confirmed pneumonia, and secondary endpoints were culture-confirmed invasive pneumococcal disease and hospital admissions. Surveillance for radiological pneumonia, invasive disease, and serious adverse events took place 7 days a week at Basse and Bansang health facilities, with referral of children from outlying clinics over dirt roads and river crossings, and each child was visited at home every 3 months for demographic surveillance.

## The Importance of a Quality Management Plan

The need for a quality management plan is recognized as part of GCP, and we developed a priority list of indicators for quality assurance (Table 1). We aimed for 100% compliance with indicators relating to following SOPs for recruitment, storage of vaccines and placebo, administration of either vaccine or placebo (which were identical in appearance and provided in numerically coded vials) correctly according to the code indicated in the randomization scheme, reporting and follow-up of serious adverse events, and investigation of children. A single error in any of these areas was discussed immediately with the relevant staff. For other indicators (e.g., dropout between first and third doses of vaccines; intervals between doses; correlation between nurses and doctors' clinical findings), we aimed for at least 95% compliance and reviewed performance at weekly staff meetings.

**Table 1:**
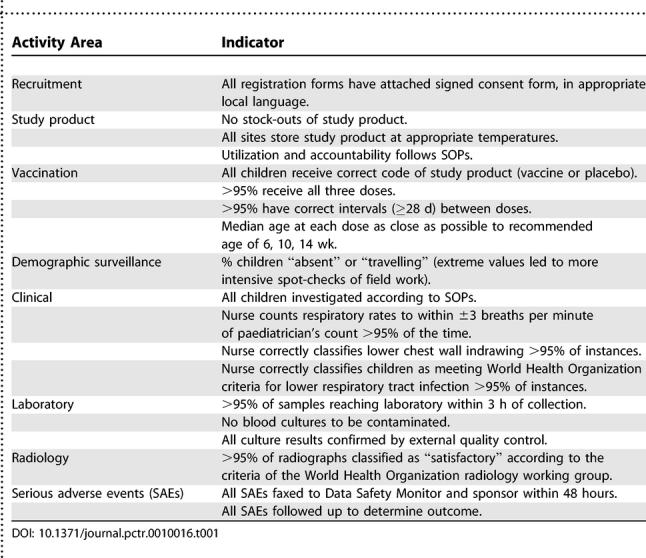
Major Indicators Used for Quality Assurance

## The Importance of On-Site Supervision and Continuous Feedback of Adherence to GCP

We monitored quality by continuous on-site observation. We used TempTale cold chain monitors and maximum-minimum thermometers to monitor vaccine storage. All completed case report forms (CRFs) were checked by field supervisors working with the epidemiologist and principal investigator (PI), with a spirit of competition as to ability to spot mistakes. We queried the data system regularly to identify the number of errors made by each person, and we conducted random spot-checks of field work and home visits. The initial procedure for supervisory rounds stipulated that supervisors visit field workers at weekends (when no recruitment or vaccination was in progress) to collect the week's CRFs, and included few details on checking actual field activities. We changed this so that supervision took place during clinic hours, and trained supervisors to observe practices and complete checklists that included the key quality indicators. To help field workers spot their own mistakes, we conducted refresher training every 2–3 months, with written tests on the SOPs, and on dummy completed CRFs on which deliberate mistakes had been made (e.g., putting a date of vaccination before a date of birth). We thus identified staff who had difficulty in noticing range and consistency checks, and gave them further on-the-job training. To improve the clinical classification of the sick child, paediatricians worked with small groups of three to four nurses at a time, each completing a one-page “quality control” form to record respiratory rates and the presence/absence of lower chest wall indrawing simultaneously and compare findings. We organized external quality control for the key endpoints, all positive pneumococcal cultures being confirmed in expert laboratories and a random sample of radiographs being read by a World Health Organization panel of radiologists.

Feedback through on-the-spot discussion of supervisors' findings, weekly staff meetings, and monthly written reports to all staff and external collaborators was done with the aim of ensuring that everyone understood the need to follow SOPs and meet GCP requirements. We incorporated relevant indicators into staff appraisals to further demonstrate the importance of quality assurance. We found that continuous checking and feedback was required throughout the study in order to prevent standards from dropping. For example, contamination of blood cultures was high at the beginning of the trial but was greatly reduced when we initiated weekly reports from the laboratory to the PI on the number of contaminated samples, and memos from the PI to the nurses who had taken those samples supplemented by discussion at clinical staff meetings each week. If we made this feedback less frequently, contamination rates tended to rise again.

Independent external trial monitors visited every 4 months to review regulatory and ethical aspects and check source documents [1]. We made it very obvious that all staff involved in the trial, from PI to field worker, were monitored. This helped to create a “culture of checking” that changed perceptions of monitoring from a threat to a management tool and overcame initial resistance to the periodic tests.

## The Importance of Documenting Roles and Responsibilities of Collaborating Groups

Five interlocking groups of personnel were involved in the trial: over 100 GG MCH staff; 150 full-time trial “clinical” staff (doctors and nurses working in three shifts to provide 24-hour cover and field workers doing home visits to the >17,000 children); 12 data staff; over 60 trial “support” staff (drivers, mechanics, administrators, clerks, cleaners, cooks, etc.), and external collaborating scientists and administrative staff based in MRC Fajara and overseas. Multiple coordination mechanisms were needed, ranging from frequent informal contacts in-person at the local level and by telephone/email internationally, to formal, minuted working groups and steering committees. Close collaboration between the trial senior management and GG health teams was important to avoid cancellation of outreach clinics that might otherwise have occurred due, for example, to shortage of transport or conflicting activities. We felt that collaboration would have been further enhanced had memoranda of understanding been written before the trial began; in their absence, it was sometimes difficult to demarcate the requirements for the research project and the wide-ranging needs of the public health system.

## The Importance of Transparent Human Resource Management Procedures

Human resource training and management took up a large proportion of time of both the PI (who focussed on quality assurance, management of clinical staff, and collaboration with external scientists) and project manager (who managed all support staff and liaised with external administrative and management personnel).

MRC's written, transparent policies on recruitment, career development, appraisal systems, disciplinary procedures, leave entitlements, health and safety, etc., helped to guide supervisors and managers (for example, by showing what procedures need to be documented in order for appropriate decisions to be made) and to set limits for negotiations between staff and managers. We revised job descriptions to ensure that they were realistic, clear, and achievable. For example, initially some job descriptions for different posts overlapped, creating the potential either for unhealthy competition or for each person thinking someone else was responsible for a particular task, whereas others were too broad to be feasible to complete. Other actions to improve staff performance included mentoring, rotating personnel between sites, and training. This ranged from distance-based learning undergraduate and postgraduate degree programmes and short courses inside and outside the Gambia, to literacy classes, basic computing, and in-service training and accreditation programmes for all categories of staff.

## The Importance of Adequate Planning for Trial Implementation

Planning for a trial requires that baseline situation assessments be conducted of the resources available for trial implementation. Given the typically long delay between planning a trial and obtaining the funding, clearances, and vaccine to begin the trial, and the time then required for recruitment and follow-up, these assessments need to predict the needs over the whole life of the trial. In our trial, adequate plans were made for purchasing cold chain equipment, but we faced substantial problems with transport and maintenance and other risks to successful completion of the trial.

### 

#### Transport.

At the trial outset, five four-wheel drive vehicles were given to the national and divisional government health teams to assist in supervision of MCH activities. In a rapid assessment of the MCH infrastructure in the study area in mid-2001, however, we found that most clinics had severe transport problems, because support to the front-line health services had not been planned. The trial therefore assigned vehicles to clinics to take MCH teams on outreach and to refer ill children to Basse or Bansang, and assisted in the maintenance of GG as well as trial vehicles. This further stretched the ability of our maintenance team to meet demands, and increased the need for close coordination between partners to prioritize vehicles and equipment for repairs. It also highlighted the need for plans to take into account the expected duration of a trial and realistic working life of vehicles in harsh conditions, and budget for replacement costs of essential items, and for written memoranda of understanding to be developed to help to manage expectations.

#### Maintenance.

Because radiological pneumonia was the primary endpoint, having access to radiology equipment was critical. The trial had refurbished radiology rooms and supplied new dryers and developing tanks, as well as bought two state-of-the-art radiology machines for Bansang hospital and Basse health centre. It proved difficult to bring engineers to maintain these in a timely way, and in 2002, we bought two portable machines for a fraction of the cost, which were more robust and produced good quality films. We maintained transport, equipment, and the all-important generators that were the source of power for the radiology equipment, laboratories, divisional cold stores, Basse health centre, and field station, and little assistance was available in this remote location. The engineering background of the project manager was therefore frequently called on, and substantial external support was required. Detailed inventories of equipment and fixed assets were developed, and use of stocks (e.g., spare parts, fuel, drugs) was computerized and monitored regularly to avoid stock-outs of critical items and reduce excessive use.

#### Risk management.

**Figure 1:**
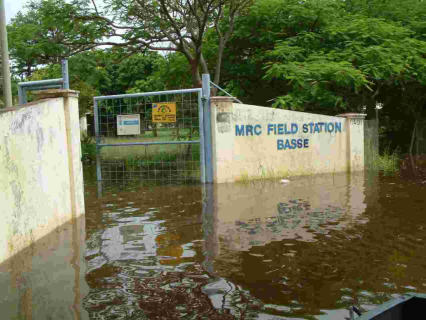
Main Gate into the MRC Field Station during the Floods Photo by Fred Yallop.

No matter how good the planning and management of trials, risks will remain. Some of the “unforeseen” risks are so common in low-resource settings that they should be expected. Those that we faced included currency devaluation, natural disasters (flooding of Basse town and field station) (see Figure 1), health and safety hazards such as road traffic accidents, national campaigns (on different occasions for measles, meningitis and polio vaccination), and deterioration in access to the study site. We needed to liaise closely with national, regional, and international authorities and identify local and external sources of support to reduce disruption of activities to a minimum.

## Conclusions and Lessons Learned

There are many operational challenges in running large field trials and strong leadership and teamwork is essential to confront them. We summarise the main ways to improve trial management in Box 1. To train personnel and generate the team spirit to implement a large trial to GCP in difficult field conditions, a senior principal investigator needs to be permanently on-site to organize quality assurance and ensure effective communication between all the groups involved, and a full-time, senior project manager is needed to organize and oversee all the support services needed to keep the trial running. We found other factors helping to achieve a high level of motivation and team spirit were the appointment of staff with appropriate and complementary skills, substantial training, objective and transparent monitoring procedures, feedback to each staff member that their work was important for the trial and for society, and recognition of their achievements by both senior staff and external advisory committees. Training is not just important at the start of a trial, it is a continuous need, because staff change and existing staff need constant motivation and support. We recommend the frequent use of written tests to assess knowledge of SOPs and ability to spot mistakes on CRFs, in addition to observation of ongoing practices. We also recommend setting out detailed and appropriate job descriptions, and including measurable performance objectives in staff appraisals. Experienced staff should act as mentors for new staff, and staff rotation between areas of work and locations can help to avoid boredom or a feeling of isolation of staff working in the more remote locations. A comprehensive quality assurance plan is vital and a mixture of internal and external monitoring and auditing is important. Visits from external monitors should be used not only for GCP audit but also to educate all staff of the reasons for, and importance of, quality control.

**Box 1. Ways to Improve the Operation of Large Phase III Field Trials**

1. All senior staff to be on-site, including PI and project manager, who must create strong team spirit.
2. Establish clear SOPs, define process indicators, and set up internal and external monitoring systems for clinical, data, and support procedures.
3. Document roles and responsibilities of collaborating groups through memoranda of understanding.
4. Define tasks clearly and realistically, assign responsibility and accountability, and make sure people know what to do and have the skills and support to do it.
5. Make plans based on situation assessments conducted by a team including experienced managers, including risk management plans.
6. Identify the appropriate resources for the trial and for partners involved in implementing the trial.
7. Take into account the operational life of transport and equipment in the relevant field conditions and budget for replacement costs of essential items.
8. Make adequate arrangements for maintenance, including skilled staff, workshops with appropriate space and safety arrangements, and timely external support as required.
9. Monitor everything closely, anticipate problems, and react early.
10. Check, check, check, check, check…and check again.

Multiple stakeholders are likely to be involved and may pool resources to run large trials. Memoranda of understanding should be written at the outset, to delineate clearly the roles and responsibilities of different partners, as well as the resources provided to or by each partner and their disposition at the end of the trial. The resources available at the study site should be reviewed carefully to determine the need for external specialist advice and identify appropriate sources for this. Aspects likely to require external support when a trial is run in a remote setting include power supplies, communications, data management (we will describe this in a separate report), cold chain, health and safety, and security.

The scientific objectives of a trial need to be translated into a detailed project development plan that outlines the key steps, activities, milestones, and critical path to ensure that trial implementation proceeds as planned. Tools such as MS Project are useful to track the multiple interlocking activities and ensure that milestones are reached on time. Training on effective project planning and evaluation in biomedical research and the use of such tools are now available via the Special Programme for Research and Training in Tropical Diseases (e.g., see www.who.int/tdr/publications/publications/training_manual.htm).

Existing clinical trial and data management guidelines should be expanded to incorporate monitoring of these areas and of accounting and budgetary management and control procedures. The management challenges in implementing large trials in resource-poor settings should not be underestimated and budgets must include adequate investment to meet these challenges. 

## Abbreviations

CRF: case report form
GCP: Good Clinical Practice
GG: Gambia Government
MCH: mother-child health
MRC: Medical Research Council
PI: principal investigator
SOP: standard operating procedure
